# Supplementary material for: Triglyceride-Glucose Index in Non-Diabetic, Non-Obese Patients with Obstructive Sleep Apnoea
Source: J Clin Med. 2021 Apr 29;10(9):1932. doi: 10.3390/jcm10091932 (PMC8125770; doi:10.3390/jcm10091932)
Supplement: Supplementary file 1 [file jcm-10-01932-s001.zip › jcm-1190402-supplementary.pdf]

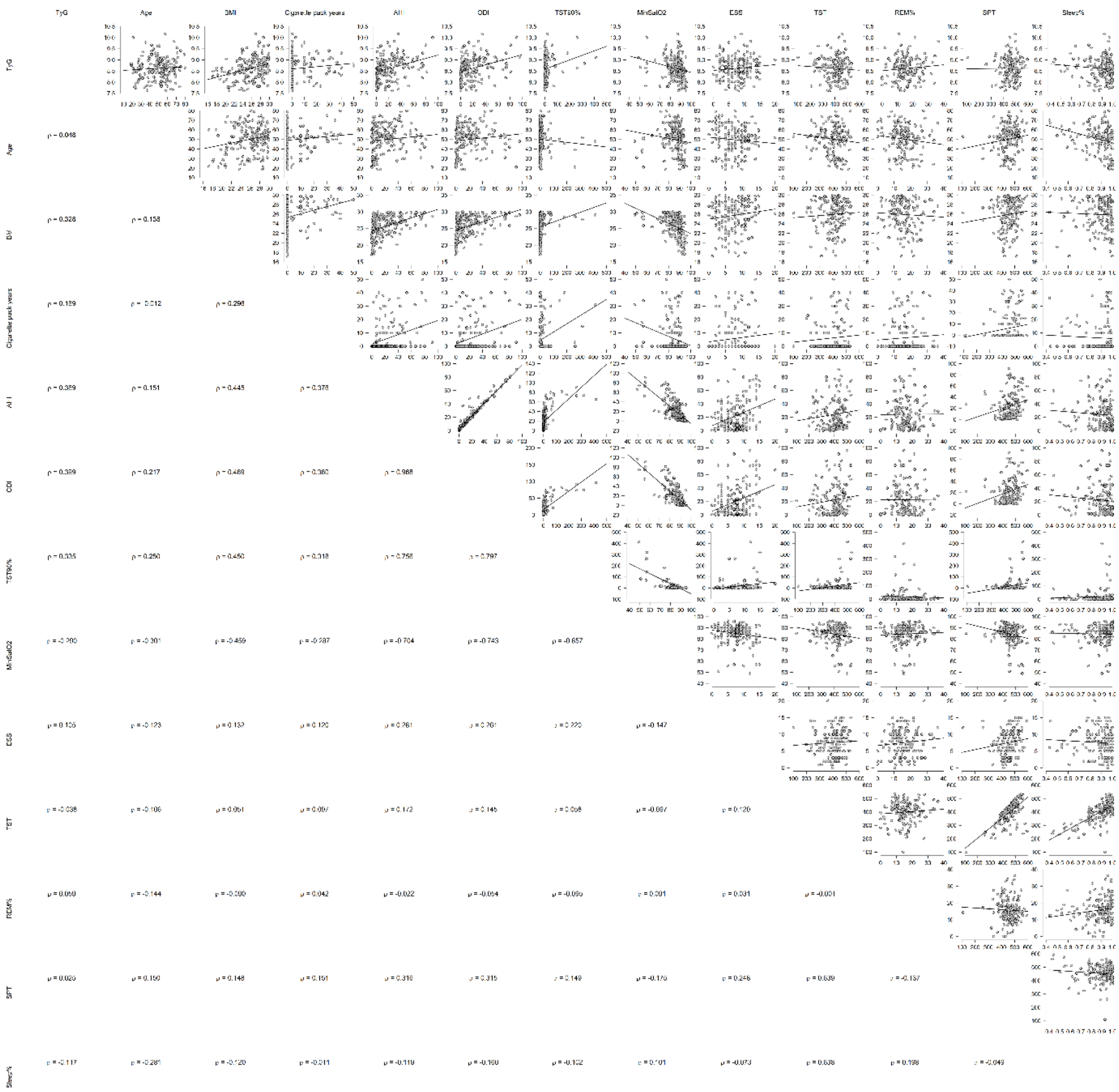

Supplementary Figure S1. Correlogram for the bivariate comparisons. AHI – apnoea-hypopnoea index, BMI – body mass index, ESS – Epworth Sleepiness Scale, MinSatO2 – minimal oxygen saturation, ODI – oxygen desaturation index, REM% – percentage of total sleep time spent in rapid eye movement sleep, Sleep% - sleep efficiency, SPT – sleep period time, TST – total sleep time, TST90% - percentage of total sleep time spent with oxygen saturation below 90%, TyG – triglyceride-glucose index
